# Length Is Associated with Pain: Jellyfish with Painful Sting Have Longer Nematocyst Tubules than Harmless Jellyfish

**DOI:** 10.1371/journal.pone.0135015

**Published:** 2015-08-26

**Authors:** Ryuju Kitatani, Mayu Yamada, Michiya Kamio, Hiroshi Nagai

**Affiliations:** Department of Ocean Sciences, Tokyo University of Marine Science and Technology, Minato-ku, Tokyo, Japan; University of Messina, ITALY

## Abstract

A large number of humans are stung by jellyfish all over the world. The stings cause acute pain followed by persistent pain and local inflammation. Harmful jellyfish species typically cause strong pain, whereas harmless jellyfish cause subtle or no pain. Jellyfish sting humans by injecting a tubule, contained in the nematocyst, the stinging organ of jellyfish. The tubule penetrates into the skin leading to venom injection. The detailed morphology of the nematocyst tubule and molecular structure of the venom in the nematocyst has been reported; however, the mechanism responsible for the difference in pain that is caused by harmful and harmless jellyfish sting has not yet been explored or explained. Therefore, we hypothesized that differences in the length of the nematocyst tubule leads to different degrees of epithelial damage. The initial acute pain might be generated by penetration of the tubule, which stimulates pain receptor neurons, whilst persistent pain might be caused by injection of venom into the epithelium. To test this hypothesis we compared the lengths of discharged nematocyst tubules from harmful and harmless jellyfish species and evaluated their ability to penetrate human skin. The results showed that the harmful jellyfish species, *Chrysaora pacifica*, *Carybdea brevipedalia*, and *Chironex yamaguchii*, causing moderate to severe pain, have nematocyst tubules longer than 200 μm, compared with a jellyfish species that cause little or no pain, *Aurelia aurita*. The majority of the tubules of harmful jellyfishes, *C*. *yamaguchii* and *C*. *brevipedalia*, were sufficiently long to penetrate the human epidermis and physically stimulate the free nerve endings of Aδ pain receptor fibers around plexuses to cause acute pain and inject the venom into the human skin epithelium to cause persistent pain and inflammation.

## Introduction

Jellyfish are classified as a member of the phylum Cnidaria. All cnidarians have a stinging cell, cnidocyte, and its stinging organelle nematocysts. A jellyfish has various nematocysts that are morphologically and functionally distinct [1], and have been classified into more than 30 types [2]. It was reported that the tubule compositions of nematocyst of jellyfish are related to their eating habits [3]. When a jellyfish tentacle touches the skin of other animals, the nematocyst discharges the tubule to sting the skin of the animal and inject the venom into its skin tissue. Both physical and chemical stimuli trigger nematocyst discharge [4, 5]. The discharge of nematocysts is one of the fastest known movements that occurs in the animal kingdom. The discharging process, in which a tubule completely everts out of a nematocyst, takes place within only 3 ms [6]. It has been speculated that this spectacular exocytosis is driven by a considerable amount of energy, which is generated by intrinsic force and osmotic pressure [7].

A large number of humans experience jellyfish stings worldwide, particularly along coastal areas of oceans. The sting is venomous and sometimes causes severe damage to humans. In an ecological context, jellyfish sting other animals when they capture prey and deter predators [1]. Although humans are neither a major predator nor prey of jellyfish, we experience a sting when accidental contact is made with a jellyfish. The symptoms caused by jellyfish stings are acute pain, followed by persistent pain and swelling of skin [8].

Jellyfish stings cause diverse types of pain and symptoms. Stings by some venomous jellyfish species are painful, whereas some from harmless jellyfish species cause subtle or no pain when they contact human skin. For example, the stings of *Chironex yamaguchii* (synonym *Chiropsalmus quadrigatus*) [9] causes severe acute pain, edema, vesiculation, painful muscular spasms, and in severe cases, dermal necrosis, the development of a rapid weak pulse, prostration, pulmonary edema, vasomotor failure, respiratory depression, and even death [10]. Three fatal cases by *C*. *yamaguchii* sting have been officially reported in Okinawa costal water in Japan [11]. *Carybdea brevipedalia* (synonym *Carybdea rastoni*) [12] has been discussed as species causing stings in pacific costal water in Japan [13]. The sting of *C*. *brevipedalia* causes severe pain, erythema, wheals, papulovesicular lesions, and slight pigmentation [13]; however, no fatalities caused by stings from this species have been reported. *C*. *yamaguchii* and *C*. *brevipedalia* sting were regarded as strong impact on public health in Japan. A species with cosmopolitan distribution, *Aurelia aurita*, inhabits areas near the shore. Despite many human encounters with *A*. *aurita*, few reports of *A*. *aurita* stings have been reported. *Chrysaora* species produce a mild-to-moderate sting that is characterized by immediate cutaneous pain, which fades over time [14]. The sting of *Chrysaora pacifica* (synonym *Chrysaora melanaster* habitat in Japanese costal water) [15] is more painful than that of *A*. *aurita*, but not as painful as that of *C*. *yamaguchii* and *C*. *brevipedalia* [16]. Furthermore, the hazard posed to humans by *C*. *yamaguchii*, *C*. *brevipedalia*, *Chrysaora quinquecirrha*, and *A*. *aurita* stings have been classified as severe, severe, moderate, and slight, respectively [17]. Therefore, in this study, we defined *C*. *yamaguchii* and *C*. *brevipedalia* stings as harmful (severely painful), *C*. *pacifica* as moderately harmful (moderately painful), and *A*. *aurita* as harmless (painless).

Studies on the venomous compounds isolated from the nematocysts have been performed [18, 19]. However, to the best of our knowledge, the mechanism through which the jellyfish sting causes pain has not yet been explored or explained.

Here, we propose that the acute pain experienced following a jellyfish sting might be generated by tubule penetration, which stimulates pain receptor neurons. In such cases, deeper penetration with longer tubules induces more severe pain. The subepidermal nerve plexus has nociceptors that mediate pain and is located 100–200 μm from the surface of the skin [20]. It is conceivable that painful jellyfish stings depend on the length of its nematocyst tubule being longer than 200 μm, leading to stimulating of pain receptor dendrites. In other words, it is possible that harmful jellyfish have longer tubules (needle) than harmless jellyfish. In this study, to test this hypothesis, we compared the lengths of the discharged nematocyst tubules from harmful and harmless jellyfish species from Japanese coastal waters.

## Materials and Methods

### Ethics statement

No specific permits were required for the described field studies. No specific permissions were required for these locations/activities. The field studies did not involve endangered or protected species.

### Animals

*Aurelia aurita* were collected from Tokyo Bay between April and May 2012. The bell diameters of the animals were between 17 and 25 cm. The tentacles were excised from living *A*. *aurita* on site, immediately frozen and stored at -30°C until treatment.

*Chironex yamaguchii* were collected from coastal area of Okinawa in August 2010. Bell heights of the animals were between 10 and 13 cm. The tentacles were excised from living *C*. *yamaguchii* on site, immediately frozen, and stored at -30°C until treatment.

*Chrysaora pacifica* were collected from Tokyo Bay between April and May 2012. Bell diameters of the animals were between 16 and 22 cm. The tentacles were excised from living *C*. *pacifica* on site, immediately frozen, and stored at -30°C until treatment.

*Carybdea brevipedalia* were collected from Miura peninsula in Kanagawa Pref. in September 2009. Bell heights of the animals were between 3 and 5 cm. The tentacles were excised from living *C*. *brevipedalia* on site, immediately frozen, and stored at -30°C until treatment.

### Isolation of nematocysts from the tentacle

To ensure equal distribution of nematocysts in the test sample, the tissue of the entire tentacles was used to isolate the nematocysts from *A*. *aurita*, *C*. *pacifica*, *and C*. *yamaguchii*. Since the tentacles of *C*. *brevipedalia* were all cryopreserved simultaneously, the tentacles used for experiments were cut off from frozen tentacles. A string of tentacle was shaken vigorously for 5 min in 20 mL 1 M NaCl solution to isolate the nematocyst. NaCl solutions containing nematocyst (nematocyst suspension) were used for microscopic observation.

### Counting nematocysts and measurement of tubule length

Three to five microliters of each nematocyst suspension, diluted appropriately with distilled water, was placed on a flat glass slide, respectively. A glass cover slide was placed on the suspension and was observed under a microscope with either 200x or 400x magnification. When nematocyst discharge did not occur spontaneously under the microscope, the cover glass was pressed using the point of a stainless steel needle to stimulate nematocyst discharge. Every nematocyst (discharged/undischarged) on the flat glass slides was photographed and the number of nematocysts was counted. All of the discharged nematocysts from each jellyfish species on the slides were used to measure the tubule length.

Digital images of the discharged nematocysts were captured under a microscope (Olympus BX50, Tokyo) equipped with a digital camera (Wraycam G-130, Osaka).

The length of each nematocyst tubule was measured by counting the pixels on the digital image using image-processing software Image J [21]. To convert a pixel to its equivalent length in the metric system, a conversion factor was calculated that corresponded to 100 μm on the digital image of objective micrometer. Tubule length was calculated by tracing the segmented-line of the tubule in the digital image. The tubule length was classified into one of 20 classes, each at every 100 μm between 0 and 2000 μm. The percentage of tubules in each class was calculated. Types of nematocysts were identified according to the descriptions provided by Mariscal [1] and Östman [2]. Numbers of the nematocysts and lengths of the tubules from three individuals of each species were analyzed by one-way ANOVA and Tukey's multiple comparison using the statistic program R version 2.14.1 for Mac OS X 10.5 or higher [22].

## Results

### Difference between species

Relative abundance of nematocyst composition and average length of nematocyst tubule are summarized in Table 1.

**Table 1:** Relative abundance of nematocyst composition and average length of tubule.

order	*A*. *aurita*			*C*. *pacifica*			*C*. *yamaguchii*			*C*. *brevipedalia*		
	nematocyst type	abundance	average length^a^	nematocyst type	abundance	average length^a^	nematocyst type	abundance	average length^a^	nematocyst type	abundance	average length^a^
1	AI^b^	59.5%	44.7 ± 30.2 μm	HME^c^	61.9%	133.3 ± 71.1 μm	MM^e^	99.0%	335.6 ± 105.0 μm	HME^c^	82.9%	826.4 ± 438.7 μm
2	HME^c^	35.9%	51.2 ± 32.0 μm	AI^b^	26.8%	92.0 ± 42.5 μm	lOR^f^	1.0%	256.3 μm	AI^b^	16.2%	165.3 ± 306.2 μm
3	unidentified	4.6%	34.1 ± 6.97 μm	unidentified^d^	11.3%	87.9 ± 41.4 μm	-	-	-	unidentified	0.9%	71.3 μm
total	all	100.0%	46.5 ± 30.4 μm	all	100.0%	117.1 ± 64.9 μm	all	100.0%	334.8 ± 104.7 μm	all	100.0%	712.4 ± 487.1 μm

Types of nematocyst were sorted by the relative abundance with each percentage and average length of tubule, respectively. Numbers (N) of measured tubule of *A*. *aurita*, *C*. *pacifica*, *C*. *yamaguchii* and *C*. *brevipedalia* were 131, 194, 104 and 111, respectively.

^a^ Average lengths (Mean ± SD) of measured tubules.

^b^ Abbreviation for atrichous isorhiza.

^c^ Abbreviation for heterotrichous microbasic eurytele.

^d^ As including isorhiza *o*-haploneme.

^e^ Abbreviation for microbasic mastigophore.

^f^ Abbreviation for large oval *p*-rhopaloid.

**Fig 1:**
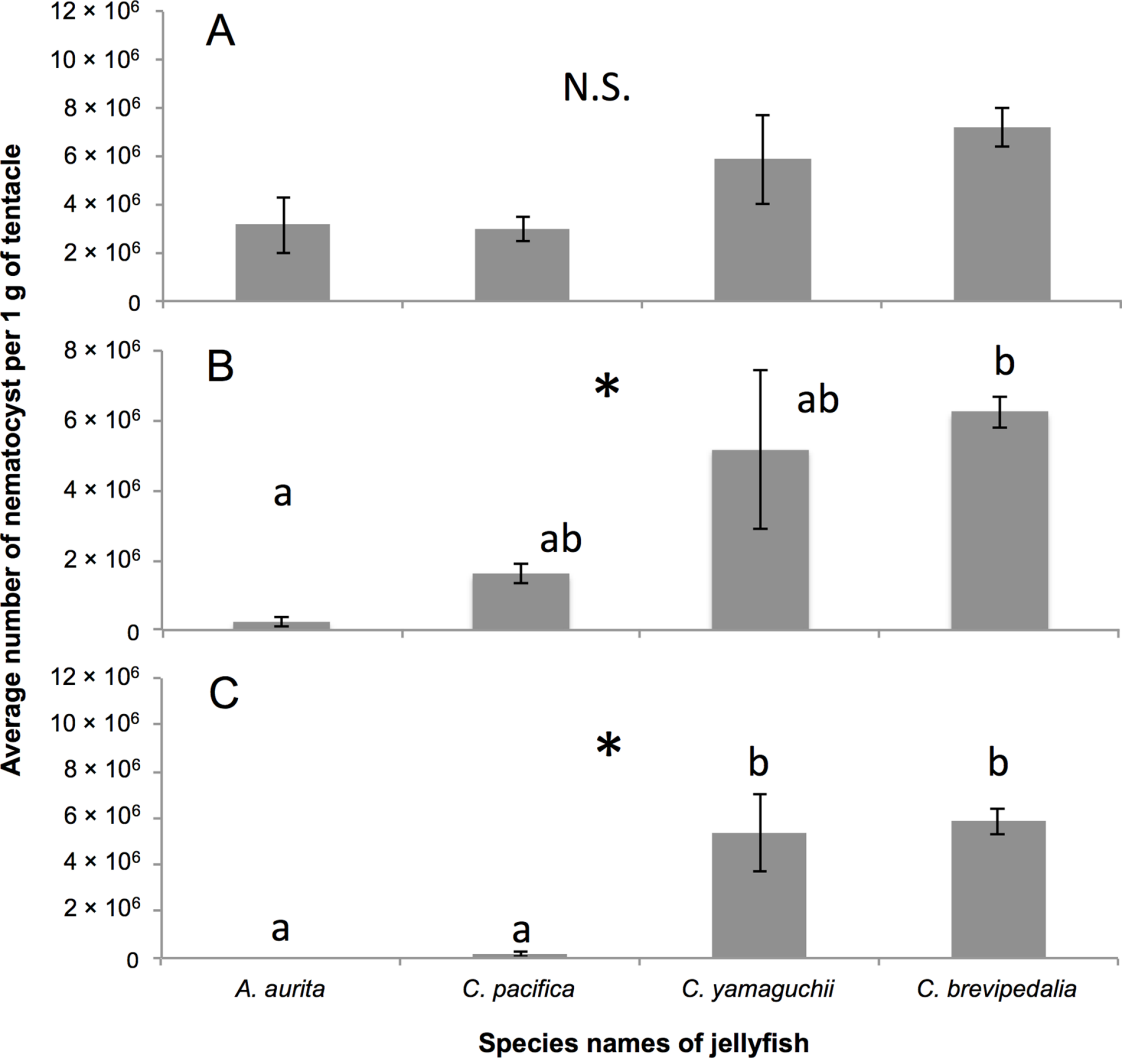
Average number of nematocyst per 1 g of tentacle. Average numbers ± SD of nematocyst in 1 g of tentacle from *A*. *aurita*, *C*. *pacifica*, *C*. *yamaguchii*, and *C*. *brevipedalia*. Ranges of tubule length are A: all, B: longer than 100 μm, and C: longer than 200 μm. N = 3 for each species. * indicates *p* < 0.05 one-way ANOVA. The same superscript letters (a, b) on the bar graph show that the bar belongs to the same group (Tukey’s test, *p*<0.05). N.S. indicates not significant.

Average numbers ± SD of nematocysts per 1 g wet tentacle from *C*. *pacifica*, *A*. *aurita*, *C*. *yamaguchii*, and *C*. *brevipedalia* were 3.01×10^6^ ± 0.74, 3.16×10^6^ ± 1.63, 5.88×10^6^ ± 2.63 and 7.22×10^6^ ± 1.15, respectively (Fig 1A). No significant differences (*F*(3,8) = 3.0, *p*>0.05) were found by one-way ANOVA in the total number of nematocysts among the four species. Comparison of the numbers of nematocysts where the tubule length was longer than 100 μm and 200μm revealed widespread differences in the numbers of nematocyst tubules in both size ranges by one-way ANOVA (*F*(3,8) = 5.9, *p*<0.05, *F*(3,8) = 13.3, *p*<0.05, respectively). Post-hoc analysis by Tukey’s method showed a difference between *A*. *aurita and C*. *brevipedalia* in the nematocyst tubules that are longer than 100 μm (*p*<0.05) (Fig 1B). In the nematocyst tubules that were longer than 200 μm, the harmless species, *A*. *aurita* and *C*. *pacifica*, were different from the harmful species, *C*. *yamaguchii* and *C*. *brevipedalia* (*p*<0.05) (Fig 1C).

**Fig 2:**
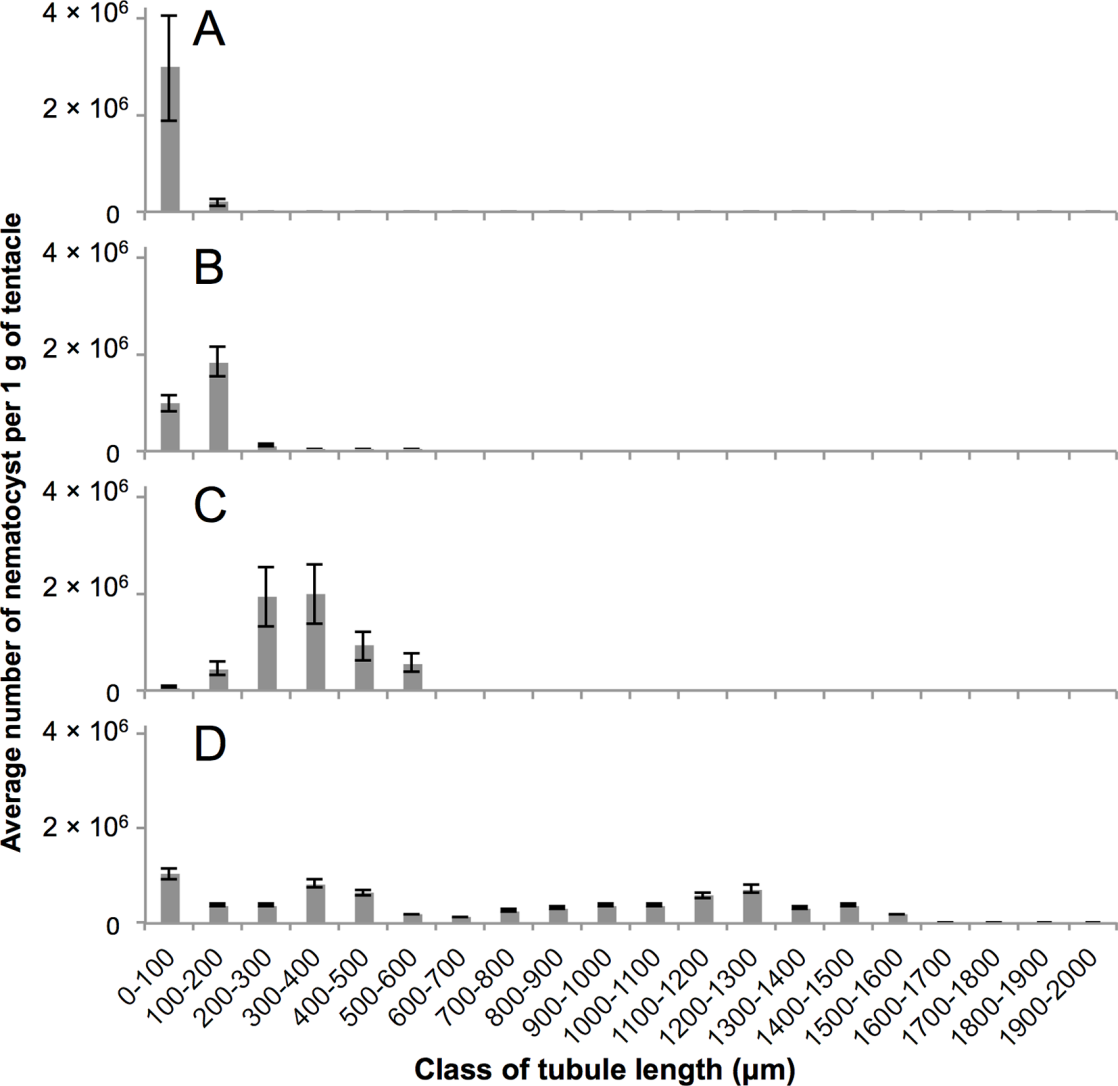
Percentage of classified length of discharged nematocyst of five jellyfishes. The measured nematocyst tubule lengths of A: *A*. *aurita*, B: *C*. *pacifica*, C: *C*. *yamaguchii*, and D: *C*. *brevipedalia* were classified into 20 classes every 100 μm. The average number ± SD of each class was plotted.

On the other hand, significant differences (*F*(3,536) = 210.8, *p*<0.05) in tubule length of total nematocyst among the four species (Table 1) were found when the data were analyzed by one-way ANOVA. Tukey's multiple comparison test was performed and significant differences (*p*<0.05) were identified in tubule length between all combinations of species. It showed that harmful species had tended to have longer tubules (*A*. *aurita* < *C*. *pacifica* < *C*. *yamaguchii* < *C*. *brevipedalia*). Distribution of the numbers of nematocyst tubules in class of tubule length is summarized in Fig 2. The average percentage of nematocysts with tubule lengths longer than 200 μm of *A*. *aurita*, *C*. *pacifica*, *C*. *yamaguchii* and *C*. *brevipedalia* were 0%, 6%, 91% and 80% (Fig 2), respectively.

### Detailed description of each species

**Fig 3:**
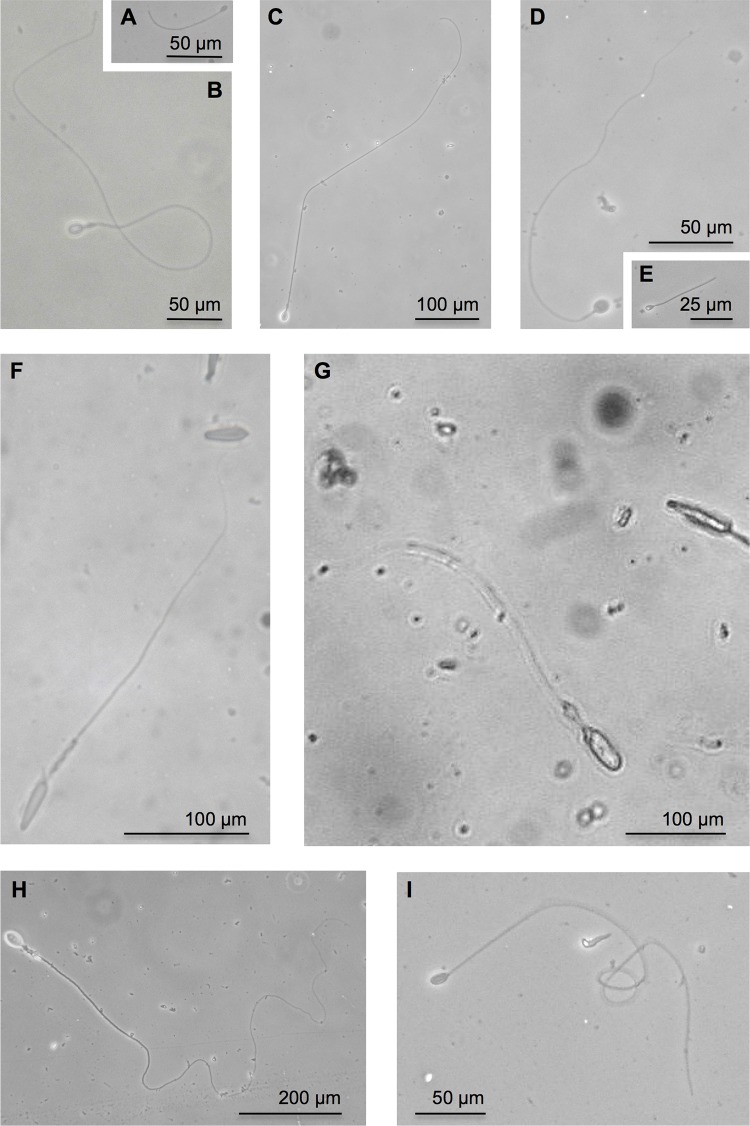
Micrographs of discharged nematocyst. The major discharged nematocysts in this study are shown. A and B were from *A*. *aurita*. C, D, and E were from *C*. *pacifica*. F and G were from *C*. *yamaguchii*. H and I were from *C*. *brevipedalia*. A) atrichous isorhiza, B) heterotrichous microbasic eurytele, C) heterotrichous microbasic eurytele D) atrichous isorhiza, E) isorhiza *o*-haploneme, F) microbasic mastigophore, G) large oval *p*-rhopaloid, H) heterotrichous microbasic eurytele and I) atrichous isorhiza. Scales are shown by the scale bar. Each nematocyst was identified by referring to previously described nematocyst nomenclature [1, 2, 23–26].

In *A*. *aurita*, 131 discharged nematocysts from three individuals were used to measure the tubule length of nematocysts. Average tubule length (± SD) was 46.5 ± 30.4 μm. (Table 1). The distribution of tubule length of *A*. *aurita* was 11.2–190.1 μm. Of the nematocysts that had their tubule length measured, 93.9% (which corresponds to 3.0 ± 1.1 ×10^6^) were present in the shortest tubule length group (0–100 μm) (Fig 2A). Out of all of the discharged nematocysts observed for *A*. *aurita*, the ratio of atrichous isorhiza (Fig 3A) and spined heterotrichous microbasic eurytele (Fig 3B) was 60% and 40%, respectively (Table 1). Nematocyst types of *A*. *aurita* were identified using the *A*. *aurita* nematocyst systematization described in previous studies [2, 23].

In *C*. *pacifica*, 194 discharged nematocysts from three individuals were used to measure the tubule length of nematocysts. Average tubule length (± SD) was 117.1 ± 64.9 μm. (Table 1). The distribution of tubule length in this species was 57.7–541.0 μm. Of the nematocysts that had their tubule length measured, 61.3% (which corresponds to 1.8 ± 0.3 ×10^6^ nematocysts) were present in the second shortest tubule length group (100–200 μm) (Fig 2B). Out of all of the discharged nematocysts observed for *C*. *pacifica*, the ratio of heterotrichous microbasic eurytele (Fig 3C), atrichous isorhiza (Fig 3D), and unidentified nematocysts including isorhiza *o*-haploneme (Fig 3E) were 62%, 27%, and 11%, respectively (Table 1). Nematocyst types of *C*. *pacifica* were identified according to the systematization described in previous studies [2, 23].

In *C*. *yamaguchii*, 104 discharged nematocysts from three individuals were used to measure the nematocyst tubule length. The average tubule length (± SD) was 334.8 ± 104.7 μm (Table 1). The distribution of tubule length of *C*. *yamaguchii* was 77.9–541.3μm. Of the nematocysts that had their tubules measured, 33.7% (which corresponds to 2.0 ± 0.6 ×10^6^ nematocysts) were present in the single tubule length group (300–400 μm) (Fig 2C). Broad local maximum was observed in the class of 200–400 μm length (Fig 2C). Microbasic mastigophore (Fig 3F) was the major nematocyst type observed in the class of 200–400 μm. Out of all of the discharged nematocysts observed for *C*. *yamaguchii*, the ratio of microbasic mastigophore and large oval *p*-rhopaloid (Fig 3G) was 99% and 1%, respectively (Table 1). Nematocyst types of *C*. *yamaguchii* were identified using the systematization previously described [24, 25].

In *C*. *brevipedalia*, 111 discharged nematocysts from more than three individuals were used to measure the tubule length of nematocysts. The average tubule length (± SD) was 712.4 ± 487.1 μm (Table 1). The distribution of tubule length of *C*. *brevipedalia* was 7.3–1524.5 μm. Of the nematocysts that had the tubule length measured, 14.4%, which corresponds to 1.04 ± 0.1 ×10^6^ nematocysts were present in the single tubule length group (0–100 μm) (Fig 2D). Almost 80% of the discharged nematocyst tubule length was over 200 μm. Out of all of the discharged nematocysts observed for *C*. *brevipedalia*, the ratio of heterotrichous microbasic eurytele (Fig 3H), atrichous isorhiza (Fig 3I), and unidentified nematocysts were 83%, 16%, and 1%, respectively (Table 1). The types of nematocysts in *C*. *brevipedalia* were identified according to the classifications previously described for *Carybdea alata* (synonym *Alatina alata*) [25, 26].

## Discussion

The type of pain and its intensity experienced following stings by jellyfish vary depending on the species [16]. In the present study, we chose four jellyfish species. *C*. *yamaguchii* and *C*. *brevipedalia* are known for their harmful stings characterised by severe acute pain. *C*. *pacifica* has a milder sting than *C*. *yamaguchii* and *C*. *brevipedalia*. *A*. *aurita* is recognized as a non-venomous, non-painful and harmless species.

According to the results of the present study, the lengths of nematocyst tubules from *C*. *yamaguchii* and *C*. *brevipedalia* jellyfish were significantly longer than those of *A*. *aurita* and *C*. *pacifica* (Fig 1). We categorize the jellyfish stings to human mentioned in the introduction into three types, according to morphological features. The first group consists in harmless species, in which most of the tubule lengths measured less than 200 μm, were represented by *A*. *aurita*. The second group consists in moderately harmful species, in which most of the tubule lengths ranged between 100 and 200 μm, were represented by *C*. *pacifica*. The third group consists in harmful species characterized by tubule lengths over 200 μm, which were represented by *C*. *yamaguchii* and *C*. *brevipedalia*. It was notable that *C*. *brevipedalia* has nematocysts with tubule lengths longer than 600 μm (Fig 2).

**Fig 4:**
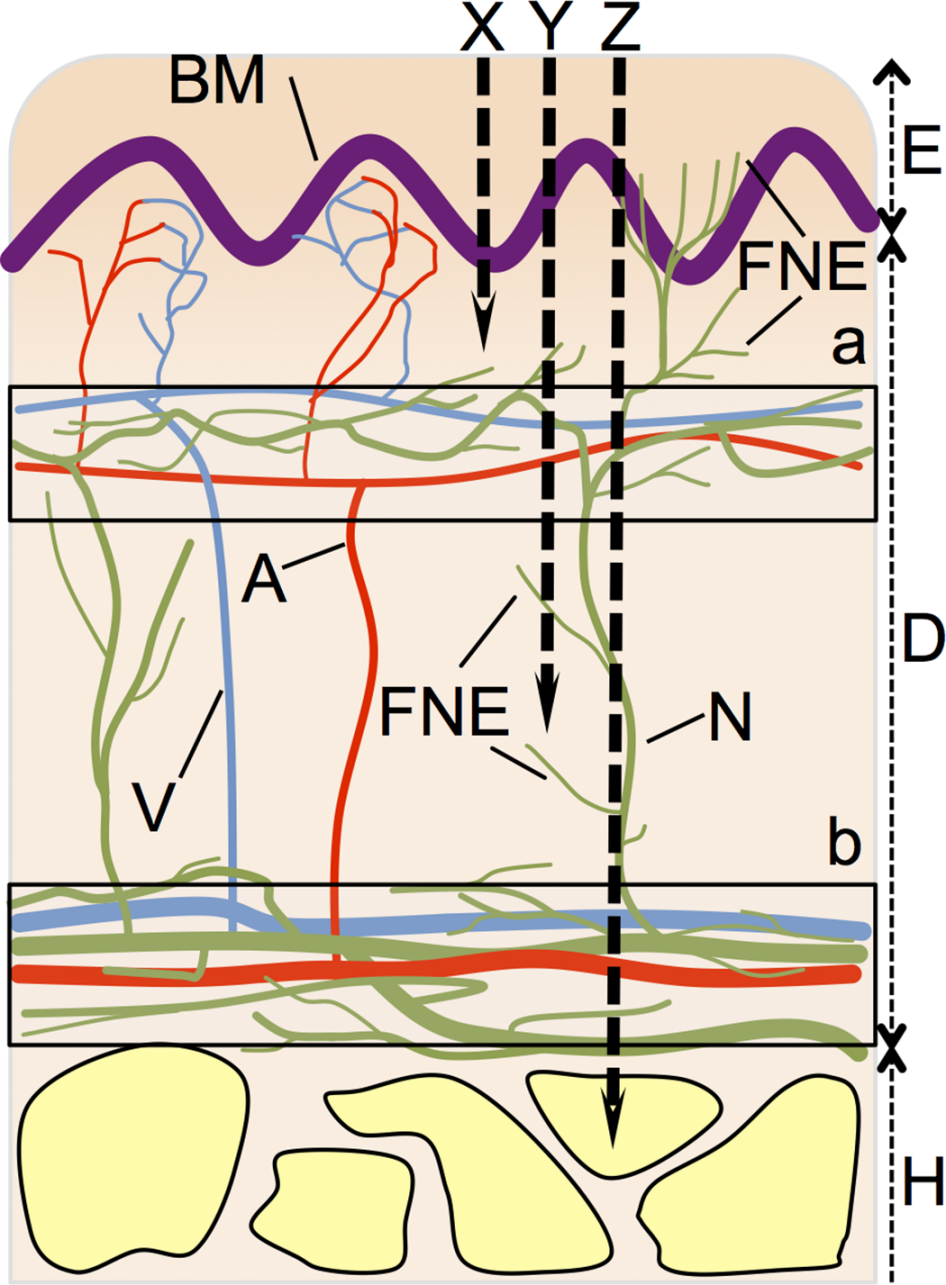
Simplified schematic of the relationship between human skin and nematocyst tubules. Simplified epidermis, dermis, and hypodermis are shown based on the reports of the biceps [20] and the lateral aspect of the upper arm [29] of human. E) epidermis, D) dermis, H) Hypodermis, A) artery, V) vein, N) nerve fiber, BM) basal membrane, FNE) free nerve ending from Aδ fiber, X) nematocyst of which tubule lengths ≤200 μm are corresponding to nematocysts of *A*. *aurita* and atrichous isorhiza of *C*. *pacifica*, Y) nematocyst of which tubule lengths from 200 μm to 600 μm are corresponding to microbasic mastigophore of *C*. *yamaguchii* and heterotrichous microbasic eurytele of *C*. *pacifica* (see Table 1), Z) nematocyst of which tubule lengths >600 μm are corresponding to heterotrichous microbasic eurytele of *C*. *brevipedalia* (see Table 1). Boxed a) Innervation range of the subepidermal nerve plexus and subpapillary plexus and boxed b) Innervation ranges of dermal nerve plexus and subcutaneous plexus.

There are some types of nociceptors that detect different types of pain and are present in primary afferent axons in human skin. It has been assumed that Aδ and C nociceptors mediate “first” and “second” pain responses, respectively, namely the rapid, acute, sharp pain and the delayed, more diffuse, dull pain evoked by noxious stimuli [27]. Afferent axons form the subepidermal nerve plexus and dermal nerve plexus in the dermis. Free nerve endings of Aδ fibers innervate the regions from plexuses that are losing myelin covering. It has been reported that Aδ fibers innervate not only the subepidermal nerve plexus, but also epidermis that is present in human skin [28]. Earlier studies showed that the subepidermal nerve plexus is located at a depth of 100–200 μm from the surface of the skin [20]. In addition, the dermis is commonly 1–2 mm thick, the shallowest distribution range of the dermal nerve plexus is at a depth of around 2 mm. Free nerve endings surrounding plexus are so dense that sensitivity to pain might increase around these areas. The distribution of nerve plexus as shown in Fig 4 is based on reports for those present in the biceps [20] and the lateral aspect of the upper arm [29] in humans.

Since the average tubule length of *A*. *aurita* nematocysts was less than 200 μm (Table 1), it is presumed they would only penetrate the epidermal layer where free nerve endings of Aδ fibers are sparse, and do not penetrate the subepidermal nerve plexus (Fig 4X). On the other hand, the major types of nematocysts in *C*. *brevipedalia* and *C*. *yamaguchii* had tubules that were longer than 200 μm (Table 1). Therefore, the tubules of *C*. *brevipedalia* and *C*. *yamaguchii* can penetrate into the region of the subepidermal nerve plexus (Fig 4Y and 4Z). These long tubules can stimulate free nerve endings in the subepidermal plexus within a few milliseconds after discharge, because the whole exocytotic process during nematocyst discharge takes less than 3 ms [6]. In fact, the stings of harmful *C*. *yamaguchii* and *C*. *brevipedalia* jellyfish cause more severe, acute pain than those of the harmless jellyfish species *A*. *aurita*. In *C*. *pacifica*, the average tubule length of nematocysts was less than 200 μm, including that of *A*. *aurita* (Table 1). However, 6% of discharged nematocyst tubules of *C*. *pacifica* were over 200 μm (Fig 2). Tubules in this class (i.e., over 200 μm) can penetrate the subepidermal nerve plexus leading to pain sensations (Fig 4Y). This might explain why the moderately harmful *C*. *pacifica* sting is more painful than the harmless *A*. *aurita* sting, and less painful than the severely harmful *C*. *yamaguchii* and *C*. *brevipedalia* stings. From these experimental results, it is plausible that tubules length is related to the acute pain which is experienced following a jellyfish sting. The subsequent persistent pain in the region of the sting might be generated due to the destruction of tissue by the deeply injected venom.

Isolation and characterization of the main proteinaceous toxins from two harmful jellyfish, *C*. *yamaguchii* and *C*. *brevipedalia*, have been reported [18, 19]. These studies showed that lethal toxicity to crayfish and hemolytic activity of the main protein toxin from *C*. *brevipedalia* is more potent than the toxin from *C*. *yamaguchii*. In the case of *C*. *yamaguchii* and *C*. *brevipedalia* stings, the deep tubule penetration into the skin should be caused by the major nematocyst type, microbasic mastigophore and heterotrichous microbasic eurytele, respectively (Table 1, Fig 3F and 3H). Nevertheless, *C*. *yamaguchii* has a main toxin of weaker activity and shorter tubule compared with *C*. *brevipedalia* (Table 1), the sting of *C*. *yamaguchii* is considered to be much more dangerous than that of *C*. *brevipedalia*. In fact, three cases of fatal stings by *C*. *yamaguchii* have been reported [11], but none by *C*. *brevipedalia* have been reported [12]. The seriousness of *C*. *yamaguchii* stings, regardless of its weak toxin and short nematocyst tubules, was discussed in a previous study [30]. Firstly, the number of *C*. *yamaguchii* tentacles is several times greater than that of *C*. *brevipedalia*. Furthermore, the length of *C*. *yamaguchii* tentacles is at least three times longer than that of *C*. *brevipedalia*. Therefore, the amount of venom that is injected with tubules into the victim by *C*. *yamaguchii* should be considerably greater than that injected by *C*. *brevipedalia* [30]. Thus, the total amount of nematocyst is another factor that affects how dangerous a jellyfish species is.

Envenomation of jellyfish sting was caused by the injection of venom with tubule penetration [31, 32]. Therefore, a nematocysts tubule of significant length could enable venom to reach the epithelium in human skin leading to cause persistent pain, inflammation, and various envenomation symptoms caused by the venom. During the discharge, venom translocates into the lumen of the tubule while the barbs emerge and extend. The venom is delivered from the everted-extended tubule through the hollow barbs into the victim [33, 34]. Various symptoms occur following envenomation by jellyfish stings. Some recent reports have indicated that the venom of *Chironex fleckeri*, which is of the same genus as *C*. *yamaguchii*, has cardiovascular effects [35–39]. Distribution of blood vessels in the human dermis is complex. The densely distributed subpapillary plexus is mainly located under the basal membrane (Fig 4a). On the other hand, the subcutaneous venous plexus close to the thicker and inner vein is mainly located above the hypodermis (Fig 4b). Therefore, deep penetration with tubules longer than 200 μm and 600 μm (Fig 2) can sufficiently cross the subpapillary plexus and subcutaneous venous plexus, respectively. Furthermore, these tubule can inject venom into the thicker and inner vein (Fig 4Y and 4Z). The effects on the cardiovascular system [40] might result in the direct injection of venom by the nematocyst tubules penetrated into the venous plexuses.

In this study, we consider the stinging tubule length to determine the acute pain experienced following a jellyfish sting. It was revealed that painful and harmful jellyfish (*C*. *yamaguchii*, and *C*. *brevipedalia*) have much longer tubules in theirs nematocysts than the moderate or none pain causing jellyfish (*C*. *pacifica* and *A*. *aurita*). The majority of the tubules of harmful jellyfish were sufficiently long (>200 μm) to penetrate the epidermis and to physically stimulate the free nerve endings of pain receptor neuron fibers causing pain and injecting venom into the human body.
